# Lrp5/6 are required for cerebellar development and for suppressing TH expression in Purkinje cells via β-catenin

**DOI:** 10.1186/s13041-015-0183-1

**Published:** 2016-01-15

**Authors:** Ying Huang, Qiong Zhang, Ning-Ning Song, Lei Zhang, Yu-Ling Sun, Ling Hu, Jia-Ying Chen, Weidong Zhu, Jue Li, Yu-Qiang Ding

**Affiliations:** Key Laboratory of Arrhythmias, Ministry of Education of China, East Hospital, Tongji University School of Medicine, Shanghai, 200120 China; Department of Anatomy and Neurobiology, Collaborative Innovation Centerfor Brain Science, Tongji University School of Medicine, Shanghai, 200092 China; Clinical and Translational Research Center, East Hospital, Shanghai, 200120 China; Department of Prevention, Tongji University School of Medicine, Shanghai, 200092 China

**Keywords:** Wnt, Lrp5, Lrp6, β-catenin, Tyrosine hydroxylase, Purkinje cell

## Abstract

**Background:**

The cerebellum is responsible for coordinating motor functions and has a unique laminated architecture. Purkinje cells are inhibitory neurons and represent the only output from the cerebellar cortex. Tyrosine hydroxylase (TH) is the key enzyme for the synthesis of catecholamines, including dopamine and noradrenaline, and it is normally not expressed in cerebellar neurons.

**Results:**

We report here that the low-density lipoprotein receptors (Lrp) 5 and 6, Wnt co-receptors, are required for the development of the cerebellum and for suppressing ectopic TH expression in Purkinje cells. Simultaneous inactivation of Lrp 5 and 6 by Nestin-Cre results in defective lamination and foliation of the cerebellum during postnatal development. Surprisingly, TH is ectopically expressed by Purkinje cells, although they still keep its other neurochemical characteristics. These phenotypes are also observed in the cerebellum of GFAP-Cre;β-catenin^flox/flox^ mice, and AAV2-Cre-mediated gene deletion leads to ectopic TH expression in Purkinje cells of β-catenin^flox/flox^ mice as well.

**Conclusions:**

Our results revealed a new role of the canonical Lrp5/6-β-catenin pathway in regulating the morphogenesis of the cerebellum during postnatal development.

**Electronic supplementary material:**

The online version of this article (doi:10.1186/s13041-015-0183-1) contains supplementary material, which is available to authorized users.

## Background

The cerebellum is located dorsal to the hindbrain and is responsible for the control of motor functions. The cerebellar cortex has a unique foliation and a 3-layer laminated architecture in which the two major cell types, Purkinje cells and granule cells, are located in the Purkinje cell layer and granule cell layer, respectively [1]. Foliation and lamination mainly occur during the early postnatal period as a consequence of drastic cell proliferation and migration. Purkinje cells are γ-aminobutyric acid (GABA) inhibitory neurons and provide the only output of the cerebellar cortex to deep cerebellar nuclei and other brain regions. They also express calcium-binding proteins parvalbumin (PV) and Calbindin, which are widely used to identify Purkinje cells [2].

Catecholamines include dopamine, noradrenaline and adrenaline, and are critical for regulating many brain functions, including memory, reward, motor control, mood, and rhythm [3]. They are expressed by specific types of neurons, such as dopaminergic neurons in the substantia nigra and noradrenergic neurons in the locus coeruleus, but not in cerebellum [3]. TH is the rate-limiting enzyme in the conversion of tyrosine to L-3,4-dihydroxyphenylalanine(L-DOPA), which gives rise to the three types of catecholamines with the help of other enzymes. For example, aromatic L-amino acid decarboxylase (AADC) is responsible for converting L-DOPA to dopamine, whereas β-hydroxylase (DBH) is required for the synthesis of noradrenaline from dopamine [4–6].

Wnt signaling is a conserved pathway in vertebrate animals. WNTs bind to and activate Frizzled and its co-receptors Lrp 5 and 6, and the downstream Wnt signaling transduction cascades include the β-catenin-dependent canonical branch and the β-catenin-independent non-canonical branches [7]. In the presence of Wnt ligands, β-catenin does not undergo degradation. Instead, it enters the nucleus and activates transcription of the Wnt target genes [8–10]. Lrp5/6 are involved in the canonical Wnt/β-catenin pathway by preventing β-catenin degradation [11, 12], and their role in inhibition of the non-canonical pathway has also been reported in our recent study [13].

Wnt signaling plays an important role in the development of the central nervous system in various aspects from early anterio-posterior axis patterning to axonal pathfinding and synaptogenesis [14–16]. The role of Wnt1 and β-catenin in the morphogenesis of the cerebellum has been examined using knockout mice [17–19], but it is still unclear if the co-receptors Lrp5 and 6 are required for the development of the cerebellum. In this study, we found that deletion of both Lrp5/6 together, but not individually, resulted in defective foliation and lamination of the cerebellum during postnatal development. Surprisingly, TH expression became ectopically present in Purkinje cells, and this phenotype was also observed in β-catenin-deficient cerebellum. Our results indicate that Lrp5/6 are required not only for the development of the cerebellum but also for suppressing TH expression in Purkinje cells possibly via β-catenin.

## Results

### Lrp5/6 dCKO but not Lrp5 CKO or Lrp6 CKO causes a defective cerebellum

To inactivate Lrp5 and Lrp6 in the cerebellum, we crossed Nestin-Cre mice [20] with Lrp5^flox/flox^ and Lrp6^flox/flox^ mice [21], and Nestin-Cre;Lrp5^flox/flox^ (hereafter referred to as Lrp5 CKO), Nestin-Cre;Lrp6^flox/flox^ (Lrp6 CKO), and Nestin-Cre;Lrp5^flox/flox^;Lrp6^flox/flox^ (Lrp5/6 dCKO) mice were obtained. Littermates with other genotypes (e.g., wild-types, Nestin-Cre with/without either Lrp5^flox/+^ or Lrp6^flox/+^) showed normal cerebellar morphology were used as controls.

**Fig. 1:**
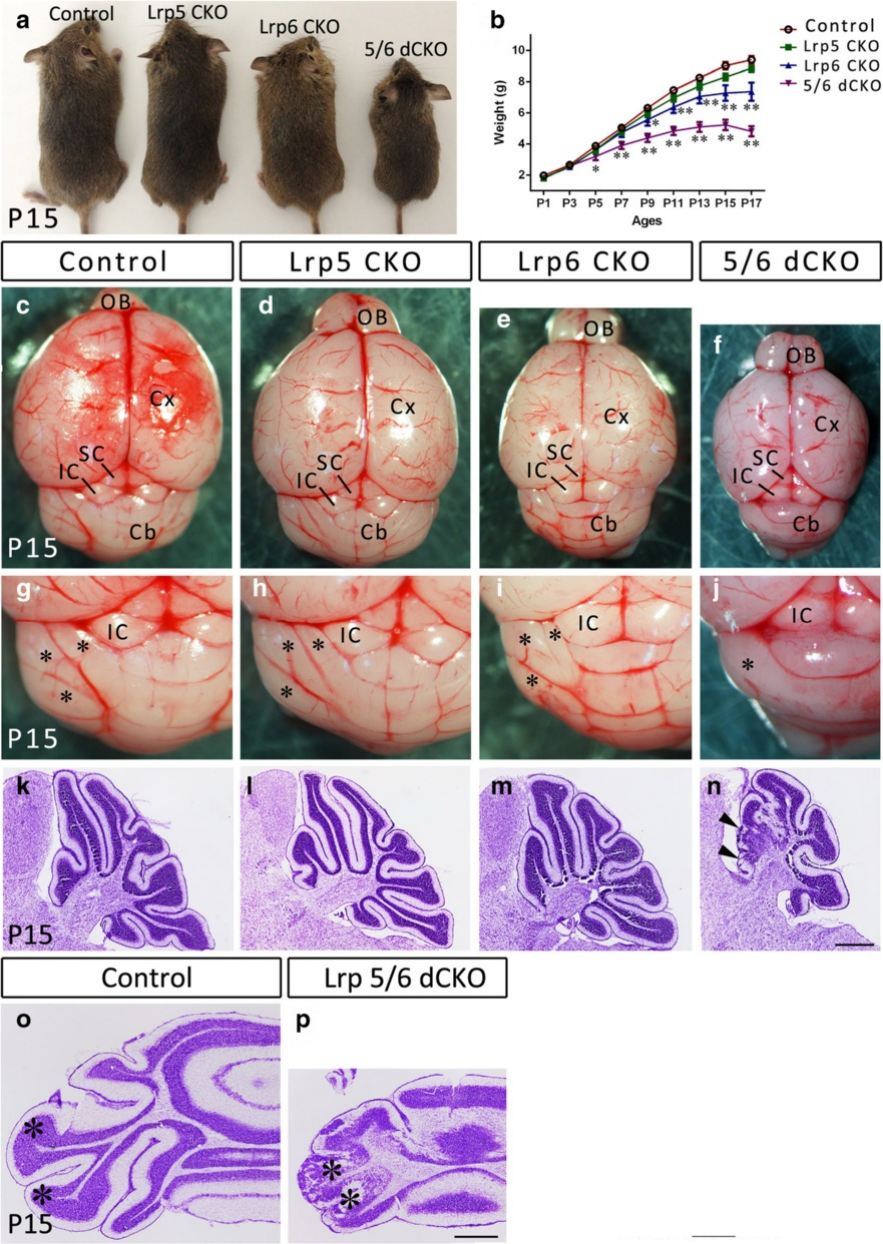
Growth retardation, defective lamination and foliation in Lrp5/6 dCKO mice. (**a**, **b**) The body sizes and body weights of Lrp6 CKO and Lrp5/6 dCKO mice are smaller than those of control and Lrp5 CKO mice. Note that Lrp5/6 dCKO mice die around P17. *p < 0.05, ** *p* < 0.01 vs WT. (**c**-**j**) Overall brain structure is maintained, but the cerebellar lobes (asterisks) in the lateral portion of the cerebellum are invisible in Lrp5/6 dCKO mice at P15. (**k**-**p**) Nissl-stained sagittal (**k**-**n**) and transverse sections (**o**, **p**) show the defective lamination and foliation in the lateral portions (asterisks, p) and anterior vermis (triangles, n) of Lrp5/6 dCKO mice at P15. Cb, cerebellum; Cx, cerebral cortex; IC, inferior colliculus; OB, olfactory bulb; SC, superior colliculus. Scale bars, 600 μm in (**n**) and applies to (**k**-**m**), and 200 μm in (**p**) and applies to (**o**)

**Fig. 2:**
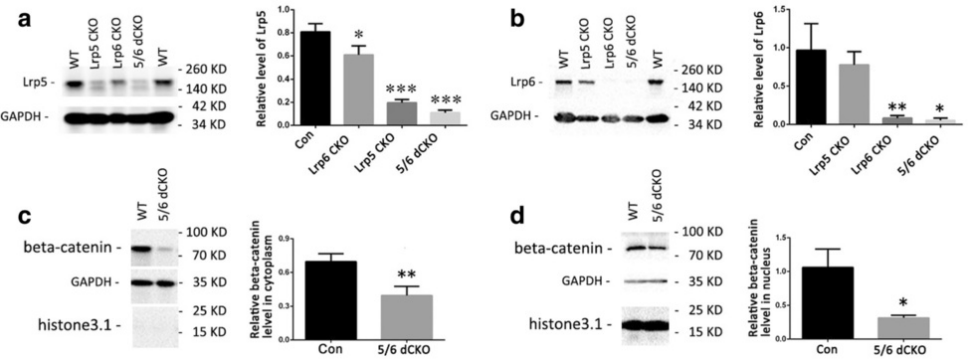
Western blot of Lrp5, Lrp6 and β-catenin in the cerebellum of CKO mice. (**a**, **b**) The cerebellar Lrp5 and Lrp6 protein levels in P17 WT, Lrp5 CKO, Lrp6 CKO, and Lrp5/6 dCKO mice. *p < 0.05, **p < 0.01,*** *p* < 0.001, vs WT control. (**c**, **d**) β-Catenin levels in the cerebellar cytoplasm (**c**) and in the nucleus (**d**) are reduced in Lrp5/6 dCKO mice. * *p* < 0.05, ** *p* < 0.01, vs WT control. Molecular weight is indicated

Lrp5 CKO and Lrp6 CKO survived into adulthood, but Lrp5/6 dCKO died during the weaning period and exhibited a reduced body weight. Lrp6 CKO mice also exhibited a reduced body weight relative to wild-type and Lrp5 CKO mice (Fig. 1a, b). Deletion of these genes was confirmed by western blot (Fig. 2a, b). We noticed that Lrp5 level was reduced in Lrp6 CKO mice, whereas Lrp6 level was not significantly changed in Lrp5 CKO mice, suggesting that normal expression of Lrp5 might be dependent on Lrp6. Macromorphological abnormalities were first observed in Lrp5/6 dCKO mice at postnatal day (P) 15 as shown by defective foliation in the lateral portion of the cerebellum (Fig. 1c-j), whereas the appearance of Lrp5 CKO and Lrp6 CKO mice was identical to control mice (Fig. 1c-j). Nissl-stained transverse sections confirmed the defective foliation in the lateral portion of the cerebellum (Fig. 1p), and sagittal sections also revealed a similar abnormality in the anterior middle portion of the cerebellum (vermis) in Lrp5/6 dCKO mice (Fig. 1k-n). These results show that a defective cerebellum is only present in Lrp5/6 dCKO mice.

**Fig. 3:**
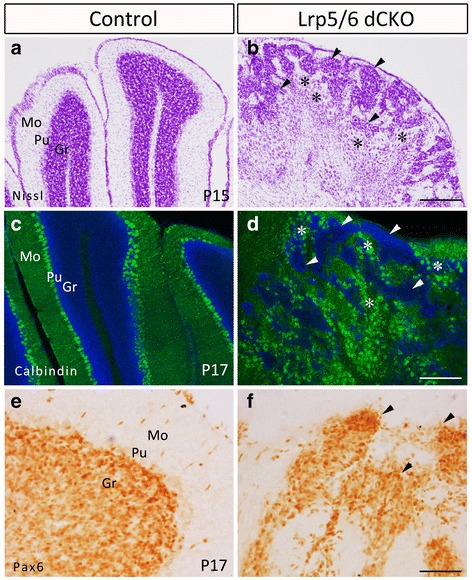
Disrupted lamination of the cerebellum of Lrp5/6 dCKO mice. (**a**, **b**) Nissl-stained P15 transverse sections showing that large cells (asterisks) and small cells (triangles) intermingle in Lrp5/6 dCKO mice, whereas they are separated into the Purkinje cell layer (Pu) and granule cell layer (Gr) in control mice. The molecular layer is clearly visible in control mice, but it is disrupted in Lrp5/6 dCKO mice. (**c**-**f**) Immunostainings of Calbindin (**c**, **d**) and Pax6 (**e**, **f**) indicate an abnormal distribution of Purkinje cells (asterisks, d) and granule cells (triangles, f) in Lrp5/6 dCKO mice compared with control mice at P17 (**c**, **e**). Mo, molecular layer. Scale bars, 120 μm in (**b**) and applies to (**a**), 200 μm in (**d**) and applies to (**c**), and 55 μm in (**f**) and applies to (**e**)

In the region with defective foliation, the laminated architecture was grossly disrupted, as shown by the presence of clusters of densely-stained small cells and weakly-stained large cells in the cerebellum of Lrp5/6 dCKO mice at P15 (Fig. 3a, b). To further confirm this, immunostaining of Calbindin, a marker for Purkinje cells, and Pax6, a marker for granule cells, was performed. Staining showed that Calbindin-positive cells and Pax6-positive cells intermingled in the cerebellum without the typical laminated architecture (Fig. 3d, f), which was clearly present in control cerebellum (Fig. 3c, e).

To find the earliest stage at which an abnormal cerebellum is evident, we performed Nissl staining and Pax6 immunostaining in Lrp5/6 dCKO mice at different postnatal stages. Nissl staining showed that there were no obvious differences at P0, but a failure of foliation was evident in the dCKO at P3 relative to control mice (Additional file 1: Figure S1a-d). The overall expression of Pax6 in Lrp5/6 dCKO mice was comparable to that in control mice at P0 and P3, although it appeared to be less evident in the external granule layer in the dCKO mice at P0 (Additional file 1: Figure S1e-h). At P7, a three-layer architecture was formed in control mice but lost in the lateral hemisphere and anterior vermis of Lrp5/6 dCKO mice, which is very similar to the phenotype observed at P15 (Fig. 3 and data not shown).

### Ectopic expression of TH in Lrp5/6 dCKO cerebellum

**Fig. 4:**
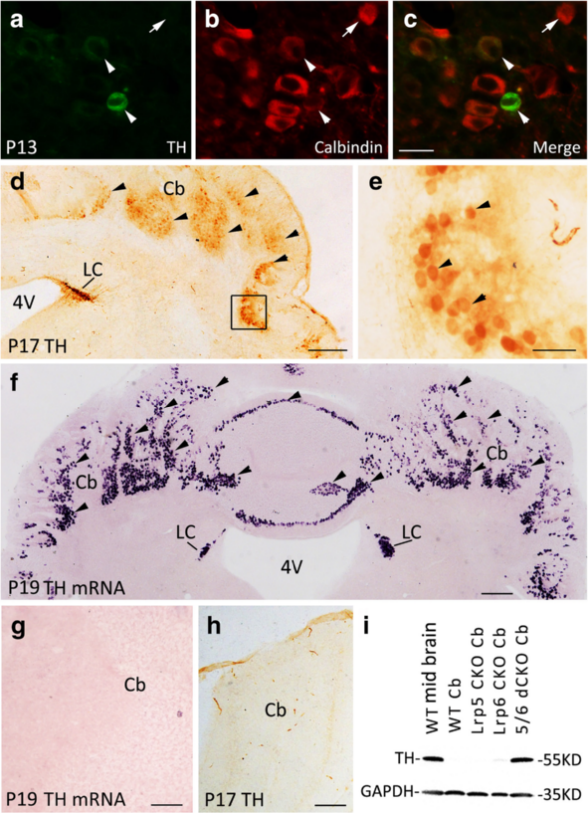
Ectopic expression of TH in the cerebellum of Lrp5/6 dCKO mice. (**a**-**c**) A few TH-positive cells are scattered in the lateral portion of the cerebellum of P13 Lrp5/6 dCKO mice, and they are Calbindin-positive. (**d**-**f**) The distribution of TH expression (triangles), shown by immunostaining (**d**, **e**) and *in situ* hybridization (**f**), is expanded, and TH-expressing cells are present throughout the cerebellum of Lrp5/6 dCKO mice at P17 (**d**, **e**) and P19 (**f**). Note that endogenous TH expression is present in the locus coeruleus (LC) (**d**, **f**). (**e**) Higher magnification of the boxed area in (**d**). (**g**, **h**) No TH transcripts or immunoreactivities are detected in WT cerebellum. (**i**) Western blot showing TH expression in Lrp5/6 dCKO mice but not in wild-type or Lrp5 CKO or Lrp6 CKO mice. Molecular weight is indicated. Scale bars, 30 μm in (**c**) and applies to (**a**, **b**), 270 μm in (**d**), 295 μm in (**f**), 100 μm in (**g**, **h**), and 40 μm in (**e**)

To explore if the neurochemical phenotype was changed by inactivation of Lrp5/6, we used a batch of antibodies to detect their expressions in Lrp5/6 dCKO brain. An unexpected finding was that many large cells were immunostained with the TH antibody throughout the cerebellum of Lrp5/6 dCKO mice at P17 (Fig. 4d, e), whereas no TH immunostaining was observed in either Lrp5 CKO, Lrp6 CKO or control cerebellum at this or earlier stages (P2, P7, P12; data not shown). As an internal control, TH immunoreactivity was observed in the locus coeruleus (Fig. 4d), which contains noradrenergic neurons. Examination of samples from different postnatal days showed that the earliest day of ectopic TH expression was observed at P13, when a small number of TH-positive cells was present in the lateral portion of the cerebellum (Fig. 4a-c). As development progressed, the number of TH-positive cells dramatically increased, and their distribution expanded throughout the Lrp5/6 dCKO cerebellum at P17 (Fig. 4d, e), whereas no immunoreactivities were observed in control mice (Fig. 4h). Western blots consistently showed TH expression in the cerebellum of Lrp5/6 dCKO but not Lrp5 CKO, Lrp6 CKO, or control mice (Fig. 4i).

We next used *in situ* hybridization to further confirm these findings. As shown in Fig. 4f, intense *in situ* hybridization signals for TH were found throughout the cerebellum of Lrp5/6 dCKO mice, and no signals were detected in control cerebellum (Fig. 4g). As mentioned earlier, the disruption of the laminated architecture was evident in the lateral cerebellar hemisphere and anterior vermis, with the exception of the posterior vermis that contained a normal 3-layer architecture (Fig. 1n, p). However, ectopic expression of TH was not restricted to the regions with disruptive lamination and was also observed in the posterior vermis (Fig. 4f), suggesting that the ectopic expression of TH is not caused by defective lamination of cerebellum. In addition, no ectopic TH immunoreactivity or mRNA was observed in other brain regions of Lrp5/6 dCKO mice. Taken together, TH is ectopically expressed in the cerebellum of Lrp5/6 dCKO mice.

### TH is expressed in Purkinje cells in Lrp5/6 dCKO

**Fig. 5:**
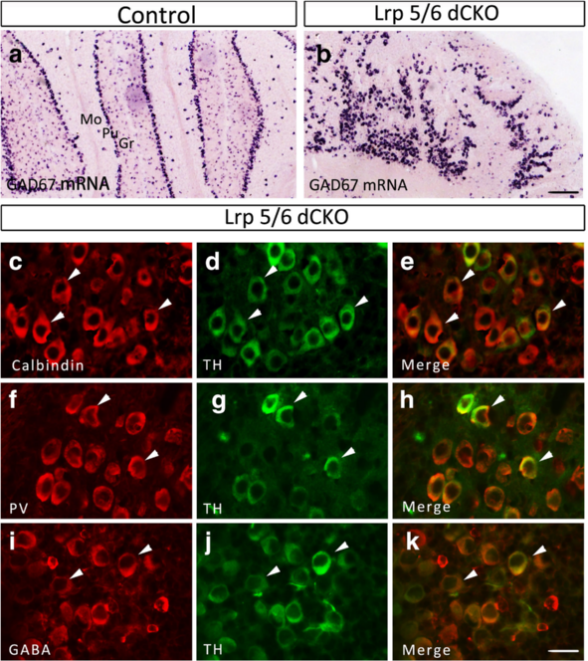
TH-expressing Purkinje cells maintain their neurochemical characteristics in Lrp5/6 dCKO mice at P17. (**a**, **b**) In situ hybridization of GAD67 showing that GABAergic neurons are primarily located in the Purkinje cell layer (Pu) in controls (**a**), but they are abnormally distributed without a one-cell layer arrangement in Lrp5/6 dCKO mice (**b**). (**c**-**k**) TH-positive cells also express Calbindin (**c**-**e**), PV (**f**-**h**), and GABA (**i**-**k**) in Lrp5/6 dCKO mice. Triangles indicate cells co-expressing TH with Calbindin, PV, and GABA. Scale bars, 200 μm in (**b**) and applies to (**a**), and 35 μm in (**k**) applies to (**c**-**j**)

Large Purkinje cells and small granule cells are the two major cell types in the cerebellum. Purkinje cells are inhibitory neurons and express Calbindin and PV [2]. TH-expressing cells in Lrp5/6 dCKO cerebellum are large in size and therefore very likely to be Purkinje cells. In addition, ectopic expression of TH suggests that the neurochemical cell fate of Purkinje cells may be altered in the absence of Lrp5/6. We thus set out to examine if these neurochemical characteristics of Purkinje cells were altered in Lrp5/6 dCKO mice. In control cerebellum, many cells in the Purkinje cell layer and a small number of them in the other two layers contained intense signals for GAD67 mRNA, whereas the distribution of cells with GAD67 mRNA was disorganized, and large GAD67-expressing cells displayed a clustered distribution pattern in Lrp5/6 dCKO mice (Fig. 5a, b). Double immunostaining showed that TH-immunopositive cells were also positive for the anti-GABA antibody (Fig. 5i-k). In addition, TH-positive cells were also labeled with Calbindin or PV immunoreactivity, although a few Calbindin- and PV-positive cells did `not express TH (Fig. 5c-h). Thus, TH is abnormally expressed by Purkinje cells but they still keep their neurochemical features in Lrp5/6 dCKO mice.

### TH-positive Purkinje cells do not differentiate into catecholaminergic neurons

**Fig. 6:**
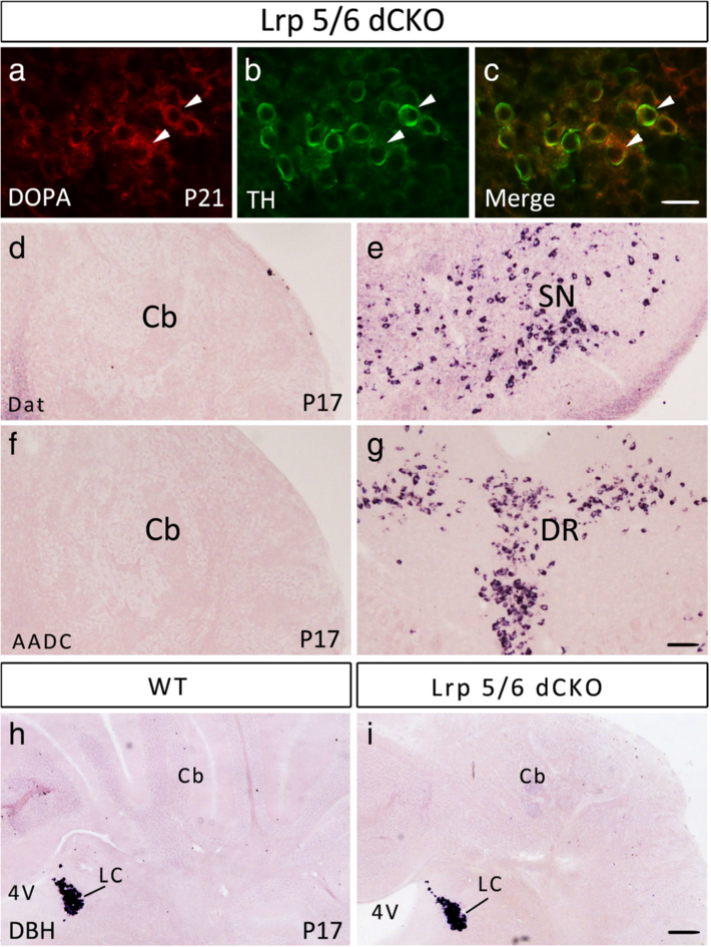
TH-expressing Purkinje cells do not differentiate into catecholaminergic neurons in Lrp5/6 dCKO mice. (**a**-**c**) TH-expressing cells are immunostained with an L-DOPA antibody in the cerebellums of Lrp5/6 dCKO mice at P21. (**d**-**g**) Cerebellar cells do not express Dat (**d**) or AADC (**f**) at P17. As an internal control, expression of Dat mRNA and AADC mRNA is observed in the substantia nigra (SN) (**e**) and dorsal raphe nucleus (DR) (**g**) in Lrp5/6 dCKO mice, respectively. (**h**, **i**) No expression of DBH in the cerebellums (Cb) of wide-type (**h**) and Lrp5/6 dCKO mice (**i**) at P17. Noted that DBH expression is observed in the locus coeruleus of both genotypes. Scale bars, 30 μm in (**a**-**c**), and 100 μm in (**d**, **f**, **h**, **i**) and 500 μm in (**e**, **g**)

Catecholamines include dopamine, noradrenaline and adrenaline. TH is responsible for catalyzing the conversion of the amino acid tyrosine to L-DOPA, the first step in the synthesis of catecholamines. L-DOPA is a precursor for dopamine, which is catalyzed by DBH to synthesize noradrenaline and adrenaline [4–6]. To examine if TH-positive Purkinje cells were dopaminergic, we performed double immunostaining of TH with L-DOPA, *in situ* hybridization of AADC, which is required for the conversion of L-DOPA to dopamine, and *in situ* hybridization of dopamine transporter (Dat), which is responsible for the reuptake of dopamine and is therefore considered to be a marker for dopaminergic neurons. We found that although TH-positive cells were immunostained with the L-DOPA antibody (Fig. 6a-c), but they did not contain either AADC or Dat transcripts in the cerebellum of Lrp5/6 dCKO mice (Fig. 6d, f), suggesting that TH-positive Purkinje cells do not differentiate into functional dopaminergic neurons. Consistently, no DBH transcripts were observed in Lrp5/6 dCKO mice (Fig. 6i). Therefore, TH-expressing Purkinje cells in Lrp5/6 dCKO are not catecholaminergic neurons.

### Ectopic expression of TH in Purkinje cells is mediated by a β-catenin-dependent pathway

**Fig. 7:**
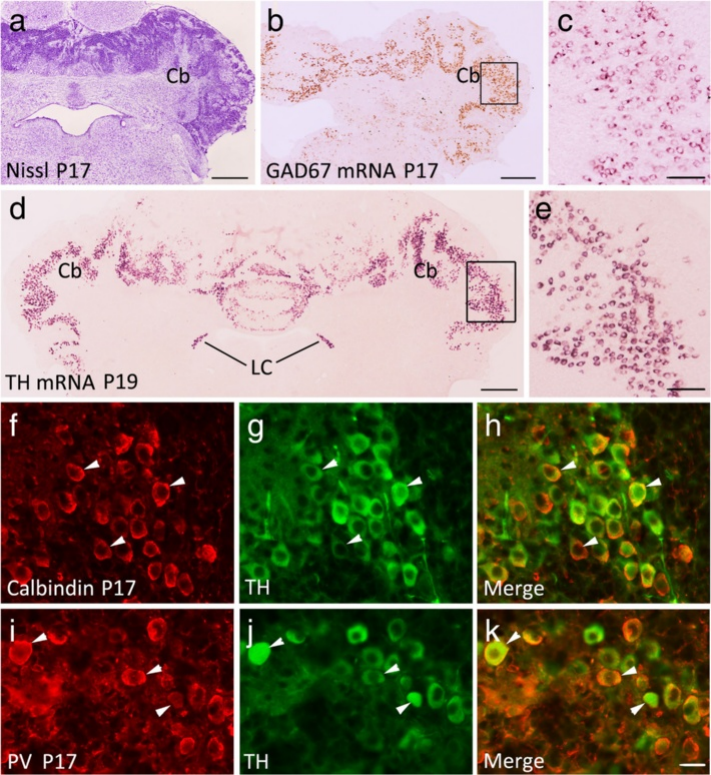
Ectopic expression of TH in Purkinje cells is also present in β-catenin CKO mice. (**a**) Disruption of the laminated architecture in β-catenin CKO mice at P17. (**b**, **c**) Many cerebellar cells express GAD67 mRNA in β-catenin CKO mice. (**c**) Higher magnification of boxed area in (**b**). (**d**, **e**) TH transcripts are abundantly distributed in the cerebellum (Cb) of β-catenin CKO mice at P19. (**e**) Higher magnification of boxed area in (**d**). (**f**-**i**) TH-immunopositive cells are immunostained with Calbindin (**f**-**h**) and PV (**i**-**k**) in the cerebellum of β-catenin CKO mice at P17. Scale bars, 670 μm in (**a**), 445 μm (**b**, **d**), 100 μm in (**c**, **e**) and 35 μm in (**f**-**k**)

**Fig. 8:**
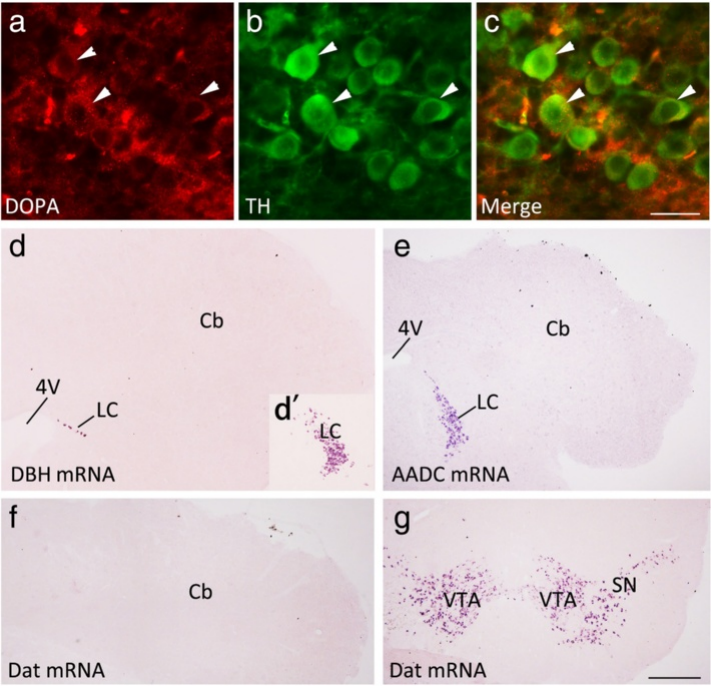
TH-expressing Purkinje cells do not differentiate into catecholaminergic neurons in β-catenin CKO mice at P17. (**a-c**) TH-expressing cells are immunostained with an L-DOPA antibody in the cerebellum of β-catenin CKO mice. (**d**-**e**) Cells do not express DBH (**d**), AADC (**e**), or Dat (**f**) in the cerebellum (Cb) of β-catenin CKO mice. Expression of DBH mRNA (**d**) and AADC mRNA (**e**) in the locus coeruleus (LC), and Dat mRNA in the substantia nigra (SN) and ventral tegemental area (VTA) (**g**) are observed in β-catenin CKO mice. (**d**’) Inset showing DBH mRNA in the LC of an adjacent section. Scale bars, 30 μm in (**c**) and applies to (**a**, **b**), and 400 μm in (**g**) and applies to (**d**-**f**, **d**’)

A previous study has shown that β-catenin is required for cerebellar lamination and foliation during postnatal development [19], which resembles the phenotypes observed in Lrp5/6 dCKO mice. We first examined β-catenin expression in Lrp5/6 dCKO cerebellum, and found significantly reduced levels in both the cytoplasm and nucleus (Fig. 2c, d). We next explored if TH is also expressed by Purkinje cells in β-catenin CKO mice (GFAP-Cre;β-catenin^flox/flox^). Consistent with a previous report [19], the 3-layer architecture was totally disrupted in the cerebellum of β-catenin CKO mice including the vermis (Fig. 7a). Many cells with intense TH transcripts were distributed throughout the cerebellum at P17 (Fig. 7d, e). Similar to Lrp5/6 dCKO mice, the first appearance of TH expression was observed around P13, as shown by scattered TH-positive neurons in the cerebellum (data not shown). Double immunostaining showed that these TH-positive cells were also co-immunostained with Calbindin and PV antibodies (Fig. 7f-k), indicating that they are Purkinje cells. In addition, GAD67-expressing cells were abundantly distributed in the cerebellum (Fig. 7b, c), and TH-positive cells were L-DOPA immunopositive but did not express AADC, DBH, or Dat in the cerebellum of β-catenin CKO mice (Fig. 8d, f). The other brain regions did not contain ectopic TH protein or mRNA in β-catenin CKO mice. Our results strongly suggest that the ectopic TH expression in Purkinje cells of Lrp5/6 dCKO mice is mediated by β-catenin.

**Fig. 9:**
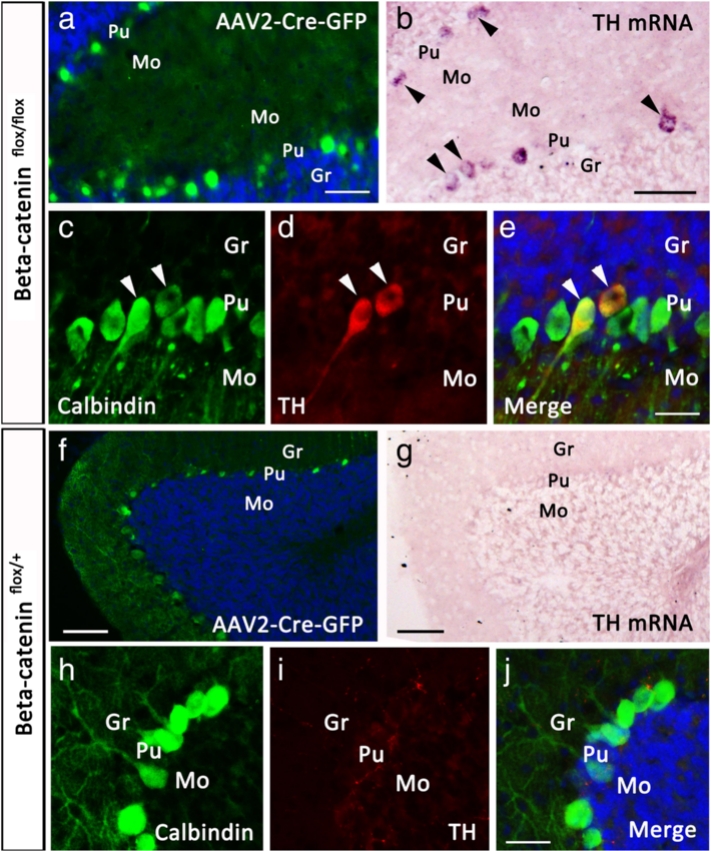
AAV2-Cre virus-mediated gene deletion also induces TH expression in Purkinje cells of β-catenin^flox/flox^ mice. (**a**-**e**) Injection of AAV2-Cre-EGFP virus into P0 cerebellum of β-catenin^flox/flox^ mice results in Cre-EGFP expression in many Purkinje cells (**a**), and some of them express TH mRNA (**b**) and possess TH immnoreactivity (**c**-**e**) at P21. (**f**-**i**) Mis-expression of Cre-EGFP does not induce TH expression in Purkinje cells of β-catenin^flox/+^ mice. Scale bars, 50 μm in (**a**), 90 μm in (**b**), 30 μm in (**e**) and applies to (**c**, **d**), 80 μm in (**f**), 70 μm in (**g**), 30 μm in (**j**) and applies to (**h**, **i**)

To further confirm this hypothesis, we used AAV2-Cre virus to delete β-catenin locally. Injection of the AAV2-Cre-EGFP virus or the control AAV2-EGFP virus into P0 cerebellum resulted in Cre-EGFP or EGFP expression in a large number of Purkinje cells at P21, but no expression was observed in granule cells (Fig. 9a, f). Injection of the AAV2-Cre-EGFP virus into cerebellum resulted in ectopic TH expression in some Purkinje cells, as shown by *in situ* hybridization (Fig. 9b) and immunostaining (Fig. 9d) in β-catenin^flox/flox^mice (Fig. 9b, d) but not in β-catenin^flox/+^ mice (Fig. 9g-i). TH expression in Purkinje cells was confirmed by double immmunostaining of TH and Calbindin in β-catenin^flox/flox^ mice (Fig. 9c-e). In addition, injection of the control AAV2-EGFP virus into the cerebellum of β-catenin^flox/flox^ mice did not induce TH expression (data not shown).

## Discussion

In this study, we deleted the Wnt co-receptors Lrp5/6 using a Nestin-driven Cre and examined the developmental phenotypes in the cerebellum of Lrp5 CKO, Lrp6 CKO, and Lrp5/6 dCKO mice. Postnatal growth retardation was observed in Lrp6 and Lrp5/6 dCKO mice, but defective formation of the cerebellum was only present in Lrp5/6 dCKO mice. In addition to defects in foliation and lamination, ectopic TH expression was observed in Lrp5/6 dCKO Purkinje cells, which was probably mediated by β-catenin.

Growth retardation was not observed in Lrp5 CKO but was more severe in Lrp5/6 dCKO relative to Lrp6 CKO mice. Nestin-Cre-mediated gene deletion is evident throughout the central nervous system, but it also occurs in other tissues (https://www.jax.org/strain/002859). Thus, the postnatal growth retardation phenotypes could not be fully ascribed to possible alterations in the brain after deletion of Lrp6 or both Lrp5 and 6. However, a defective cerebellum was only observed in the Lrp5/6 dCKO, showing that there is functional redundancy between Lrp5 and Lrp6 in regulating the development of the cerebellum.

The role of Wnt signaling in cerebellar development has been reported previously [17–19]. Wnt1 mutant mice display a loss of the midbrain and adjacent cerebellar components of the metencephalon, and these phenotypes are resulted from abnormal gene expression caused by mutation of Wnt1 during neural plate induction [17]. Pups with a Nestin-Cre-mediated deletion of β-catenin die at birth, and vermian hypoplasia and failure of fusion of the cerebellar hemispheres were reported [18]. However, because these mutants die soon after birth, it hampers investigating the role of Wnt signaling in postnatal cerebellar development, which involves drastic morphological changes including size expansion, foliation, and lamination. A study by Wen et al. [19] explored this question by conditional deletion of β-catenin using GFAP-driven Cre and reported defective foliation and lamination in the cerebellum in β-catenin CKO mice; this phenotype was confirmed in our β-catenin CKO mice. In this study, we found that defects in the cerebellar foliation and lamination were less severe in Lrp5/6 dCKO mice than in β-catenin CKO mice. For example, the laminated structure of the posterior vermis was maintained in Lrp5/6 dCKO, whereas foliation and lamination were disrupted throughout the cerebellum of β-catenin CKO mice, suggesting that an Lrp5/6-independent mechanism is involved in the function of β-catenin in regulating the development of the cerebellum, given the facts that two different Cre lines were used to knockout the different genes. Nevertheless, the canonical Wnt pathway is required for the morphogenesis of the cerebellum during both embryonic and postnatal development.

An unexpected finding of this study was that Purkinje cells in the cerebellum initiated TH expression in Lrp5/6 dCKO mice around P13. In a normal brain, TH is only expressed by catecholaminergic neurons that are located in specific brain regions but not in the cerebellum. Because of the involvement of Lrp5/6 in the canonical Wnt pathway and similar phenotypes in β-catenin CKO mice, our genetic evidence strongly suggests that Lrp5/6 deletion-induced initiation of TH expression in Purkinje cells is mediated by β-catenin. In support of this hypothesis, Western blot data showed that β-catenin levels were reduced in Lrp5/6 dCKO cerebellum. However, it is unclear how the loss of function in this pathway triggers TH transcription and translation in Purkinje cells. Transient expression of TH has been reported in several brain regions including the cerebellum during embryonic and postnatal development [22–25]. Recombination signals were consistently observed in the cerebral cortex, inferior colliculus, and cerebellum (i.e., Purkinje cells) in the adult brain of TH-Cre:Rosa26 mice [6]. However, ectopic TH expression was observed only in Purkinje cells of the cerebellum of Lrp5/6 dCKO and β-catenin CKO mice. Injection of the AAV2-Cre virus into the cerebellum resulted in Cre expression in Purkinje cells, and some of them initiated TH expression in β-catenin^flox/flox^ mice. Therefore, it might be concluded that the Lrp5/6-β-catenin pathway functions as a suppressor preventing the re-initiation of the genetic machinery responsible for TH transcription and translation in Purkinje cells during postnatal development. Further studies are needed to explore this possibility, for example whether there are any Wnt response elements in the TH promoter.

Although Purkinje cells expressed TH and contained DOPA, they lacked the other catecholamine-synthesizing enzymes (i.e., AADC and DBH) and Dat, showing that they are not dopaminergic or other catecholaminergic neurons in Lrp5/6 dCKO or β-catenin CKO mice. A previous study reported that primary sensory neurons in the dorsal root ganglia (DRG) express TH without the catecholamine-synthesizing enzymes [26]. Consistent with this report, we found that the neurochemical characteristics of Purkinje cells were maintained, as shown by the expression of GAD67, GABA, Calbindin, and PV. There are no clear answers to explain the biological significance of persistent TH expression in the DRG and transient expression in other brain regions. One possibility proposed in the previous study is that after release L-DOPA is extracellularly converted to dopamine [26].

## Conclusions

In this study, we deleted Wnt co-receptors Lrp5/6 in the brain, and found that Lrp5/6 are required for the foliation and lamination of the cerebral cortex during the postnatal development. An unexpected finding was that TH was ectopically expressed by Purkinje cells in Lrp5/6 dCKO mice. In addition, ectopical expression of TH was also observed in β-catenin CKO mice, and deletion of a-catenin by AAV2-Cre virus also resulted in TH expression in β-catenin^flox/flox^ mice. Our study indicates that Lrp5/6 are required for the morphogenesis of the cerebellum, and a new role of Lrp5/6-β-catenin pathway in suppressing ectopic TH expression in cerebellar Purkinje cells during the postnatal development.

## Methods

### Genotyping and maintenance of animals

Nestin-Cre [20], GFAP-Cre [27], Lrp5^flox/flox^ and Lrp6^flox/flox^ [21], and β-catenin^flox/flox^ mice [28] were genotyped as described previously. Lrp5 CKO, Lrp6 CKO, and Lrp5/6 dCKO mice were obtained by crossing Nestin-Cre with Lrp5^flox/flox^ and Lrp6^flox/flox^. β-catenin CKO mice were obtained by crossing GFAP-Cre mice with β-catenin^flox/flox^ mice. In each set of experiments, at least 3 CKOs and an equal or more number of control mice were used. All experiments on animals have been reviewed and approved by the Animal Research Committee of Tongji University School of Medicine, China.

### Histological analysis, immunohistochemistry, and *in situ* hybridization

Pups at different postnatal days were perfused with 4 % paraformaldehyde (PFA) in 0.01 M phosphate buffered saline (PBS; pH 7.4). PFA-fixed brains were sectioned in the sagittal or coronal plane on a cryostat after cryoprotection with 30 % sucrose in PBS. Nissl staining and immunohistochemistry were performed as described previously [29, 30]. For Nissl staining, 20-μm-thick sections were stained with Cresyl Violet. For double immunofluorescent staining, sections were incubated with a combination of mouse anti-TH (1:20,000, Sigma) and rabbit anti-Calbindin (1:2,000, Sigma), rabbit anti-PV (1:300, Swant), rabbit anti-GABA (1:1,000, Sigma), rabbit anti-DOPA (1:1,000, Sigma), or rabbit anti-DBH (1:500, Abcam) overnight at 4 °C. They were then incubated with a combination of Alexa Fluor 488-conjugated horse anti-mouse IgG (1:500, Invitrogen) and biotinylated horse anti-rabbit IgG (1:500, Vector Laboratories) for 3 h. Finally, they were incubated with Cy3-conjugated streptavidin (1:1,000, Jackson ImmunoResearch Laboratories) for 2 h at room temperature. Cell nuclei were visualized by staining with Hoechst 33258 (1:2,000, Sigma).

*In situ* hybridization was performed on 30-μm-thick sections fixed in 4 % PFA in PBS, and digoxigenin-labeled riboprobes were used to detect the expression of TH, GAD67, DBH, Dat and AADC. The signals were visualized in alkaline phosphatase buffer containing NBT and BCIP. These probes were constructed according to the descriptions on the website of the Allen Brain Atlas (http://www.brain-map.org).

### Western blot

P17 cerebellum or midbrain tissue was lysed in M-PER reagent using a protease inhibitor cocktail (Thermo Scientific). Nuclear proteins and plasma proteins were isolated using a commercial extraction kit (Sigma). Equal amounts of proteins were fractionated by SDS-PAGE, transferred to nitrocellulose membranes, and blotted with primary antibodies. The following primary antibodies were used: rabbit anti-TH (1:500, Sigma), mouse anti-β-catenin (1:2000, BD bioscience), rabbit anti-Lrp5 (1:100, Cell Signaling Pathway), rabbit anti-Lrp6 (1:500, Cell Signaling Pathway), and mouse anti-GAPDH (1:2000, Santa Cruz) or rabbit anti-Histone3.1 (1:1000, CMCTAG) was used as an internal control. After primary antibody incubation, the membranes were treated with an HRP-conjugated secondary antibody and exposed for chemiluminescent detection (Thermo Scientific). The protein levels were analyzed using Adobe Photoshop CS5 (Adobe Systems) and normalized relative to the internal control.

### Infection with adenoviruses (AAV)

AAV2-EGFP and AAV2-Cre-EGFP viruses (~6x10^12^ VG/ml) were purchased from Shanghai Taiting Biotechnology Co. Ltd, China. Postnatal virus injection was performed in P0 pups anesthetized on ice. A glass needle was connected to a manual Microinjector (CellTramvario, Appendorf), and 0.5 μl virus-containing solution was injected slowly into the cerebellum under a dissecting microscope. Pups were sacrificed at P21 for examination of TH expression as mentioned above.

### Growth curve observation

Pups, including 8 Lrp5 CKOs, 7 Lrp6 CKOs, 8 Lrp5/6 dCKOs, and 14 control littermates, were weighed every 2 days from P1 to P17. The data were recorded and then analyzed.

### Statistical analysis

All values are expressed as the mean ± SEM, and analyzed using SSPS software (IBM Inc). Comparisons were made using student’s t-test, or one-way ANOVA, followed by a LSD *post-hoc* test. P values less than 0.05 were considered statistically significant.

## Additional file

Additional file 1: Figure S1. The earliest stage of defective cerebellar morphology in Lrp5/6 dCKO mice. (a-d) Nissl staining shows that failure of cerebellar foliation is not evident in the dCKO mice at P0 (b) but is observed at P3 (d) compared to control mice (a, c). (e-h) Pax6 expression in control and Lrp5/6 dCKO mice at P0 (e, g) and P3 (f, h). EGL, external granule layer. Scale bars, 500 μm in (d) and applies to (a-c), and 25 μm in (h) and applies to (e-g). (PDF 400 kb)

## Abbreviations

AADC: L-amino acid decarboxylase
AAV: Adenoviruses
Dat: Dopamine transporter
DBH: β-hydroxylase
L-DOPA: L-3,4-dihydroxyphenylalanine
GABA: γ-aminobutyric acid
Lrp5: Low-density lipoprotein receptor 5
Lrp6: Low-density lipoprotein receptor 6
PV: Parvalbumin
TH: Tyrosine hydroxylase
