# Supplementary material for: Lrp5/6 are required for cerebellar development and for suppressing TH expression in Purkinje cells via β-catenin
Source: Mol Brain. 2016 Jan 15;9:7. doi: 10.1186/s13041-015-0183-1 (PMC4714458; doi:10.1186/s13041-015-0183-1)

Supplementary figure legends

Supplementary Figure 1 The earliest stage of defective cerebellar morphology in Lrp5/6 dCKO mice. (a-d) Nissl staining showing that failure of cerebellar foliation is not evident in the dCKO mice at P0 (b) but is observed at P3 (d) compared to control mice (a, c). (e-h) Pax6 expression in control and Lrp5/6 dCKO mice at P0 (e, g) and P3 (f, h). EGL, external granule layer. Scale bars, 500 µm in (d) and applies to (a-c), and 25 µm in (h) and applies to (e-g).

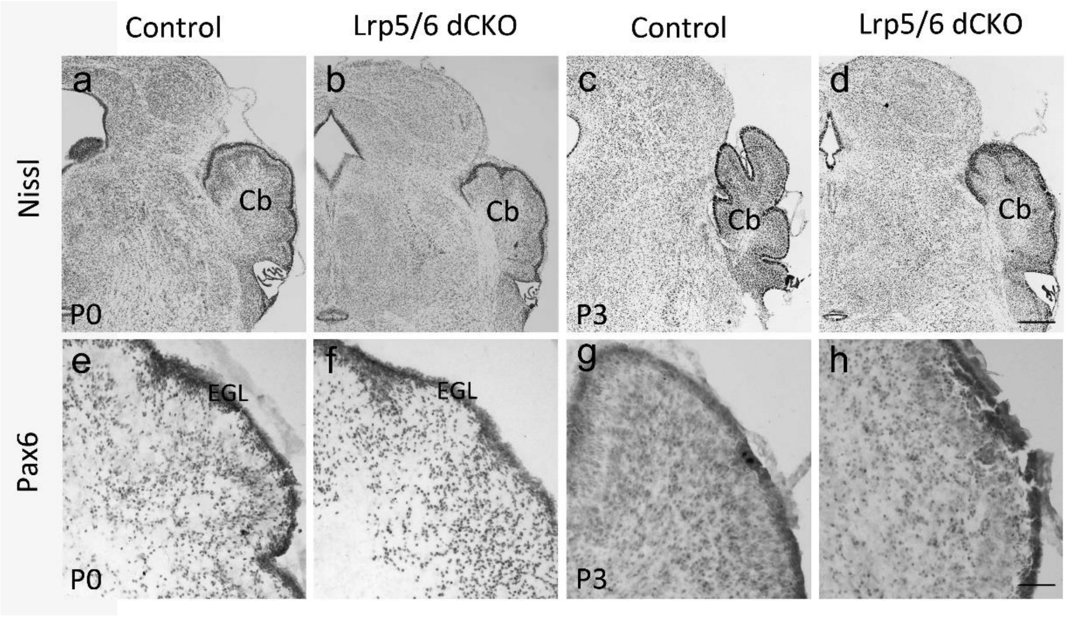

Supplement: Additional file 1: Figure S1. — The earliest stage of defective cerebellar morphology in Lrp5/6 dCKO mice. (a-d) Nissl staining shows that failure of cerebellar foliation is not evident in the dCKO mice at P0 (b) but is observed at P3 (d) compared to control mice (a, c). (e-h) Pax6 expression in control and Lrp5/6 dCKO mice at P0 (e, g) and P3 (f, h). EGL, external granule layer. Scale bars, 500 μm in (d) and applies to (a-c), and 25 μm in (h) and applies to (e-g). (PDF 400 kb) [file 13041_2015_183_MOESM1_ESM.pdf]
